# Combined Neuroprotective Effects of N,N‐Dimethyltryptamine and Ventral Root Reimplantation Following Spinal Root Avulsion in Rats

**DOI:** 10.1111/jnc.70364

**Published:** 2026-01-29

**Authors:** Paola Andrea Caro Aponte, Edison Huertas Montoya, Italo O. Mazali, Alessandra Sussulini, Benedito Barraviera, Rui Seabra Ferreira, Luciana Politti Cartarozzi, Alexandre Leite Rodrigues de Oliveira

**Affiliations:** ^1^ Laboratory of Nerve Regeneration University of Campinas (IB/UNICAMP) Campinas Brazil; ^2^ Functional Materials Laboratory Institute of Chemistry, University of Campinas (UNICAMP) Campinas Brazil; ^3^ Laboratory of Bioanalytics and Integrated Omics (LaBIOmics) Institute of Chemistry, University of Campinas (UNICAMP) Campinas Brazil; ^4^ Center for the Study of Venoms and Venomous Animals (CEVAP) São Paulo State University (UNESP) Botucatu Brazil

**Keywords:** CNS/PNS interface injuries, fibrin sealant biopolymer, motoneurons, N, N dimethyltryptamine, ventral root avulsion

## Abstract

Currently, no effective treatment exists for injuries at the interface between the CNS/PNS, largely due to their complex pathophysiology and the limited efficacy of single‐target therapies. To address this challenge, we investigated a novel combinatorial therapeutic strategy integrating surgical VRR with fibrin sealant biopolymer (FSB) and DMT in a rat model of ventral root avulsion VRA. DMT was extracted from *Mimosa tenuiflora* roots and structurally characterized using standard analytical methods. Adult female Lewis rats underwent unilateral L4‐L6 VRA and received daily DMT treatment (1, 2.5, or 5 mg/kg; i.p) for 2 weeks to determine the optimal therapeutic dose. Subsequently, the identified optimal DMT dose was combined with VRR, and animals were evaluated 2 weeks post‐injury. Outcome measures encompassed quantitative assessments of neuronal survival, glial reactivity, synaptic preservation, and differential gene expression of neurotrophic factors (GDNF, FGF‐2, VGF‐A) and anti‐apoptotic genes (Bcl‐2, Bcl‐XL). Extracted DMT met all structural and analytical criteria for experimental use. Proximal axotomy led to substantial MN loss (78%), accompanied by pronounced glial reactivity and synaptic detachment. DMT at 1 mg/kg yielded the strongest neuroprotective profile, significantly enhancing MN survival, reducing glial reactivity, and preserving pre‐synaptic boutons. Notably, these effects were further potentiated when DMT treatment was combined with VRR. Moreover, the combined VRR + DMT therapy significantly upregulated GDNF expression, indicating a synergistic effect on neurotrophic support. Overall, our findings suggest that DMT is a promising neuroprotective agent for treating MN degeneration following CNS/PNS interface injuries, particularly when integrated into a combinatorial therapeutic strategy.

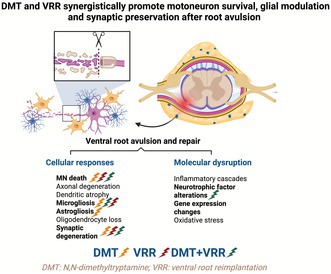

## Graphical abstract

The ventral root avulsion (VRA) model provides an excellent experimental paradigm for studying injuries at the Central/Peripheral Nervous Systems (CNS/PNS) interface. By physically disconnecting motoneurons (MNs) from the spinal cord surface, VRA induces profound cellular, molecular, and functional deficits with limited spontaneous recovery. Given the complex pathophysiology underlying this injury, combined therapeutic strategies targeting multiple mechanisms simultaneously hold considerable promise. Here, we hypothesized that N, N dimethyltryptamine (DMT) and ventral root reimplantation (VRR) administered either as monotherapies but especially in combination could ameliorate degenerative changes and improve outcomes following VRA.

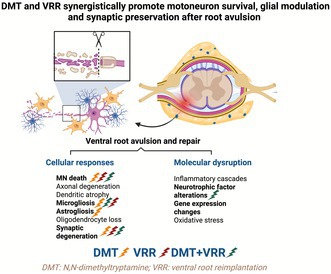

**Abbreviations**
^1^H NMR: proton nuclear magnetic resonance
BDNF: brain‐derived neurotrophic factor
CEMIB: Multidisciplinary Center for Biological Investigation of the UNICAMP
CEUA: Committee for Ethics in Animal Use
CEVAP: Center for the Study of Venoms and Venomous Animals
CNS: central nervous system
CNTF: ciliary neurotrophic factor
DMT: N, N dimethyltryptamine
FGF‐2: fibroblast growth factor 2
FS: fibrin sealant
FSB: fibrin sealant biopolymer
FTIR: Fourier‐transform infrared spectroscopy
LC‐MS/MS: liquid chromatography coupled to tandem mass spectrometry
GDNF: glial cell‐derived neurotrophic factor
GFRα: GDNF family receptor alpha‐1
MN: motoneuron
PNS: peripheral nervous system
ROS: reactive oxygen species
RRID: research resource identifier
RT: retention time
S1R: sigma‐1 receptor
VRA: ventral root avulsion
VRR: ventral root reimplantation

## 1 Introduction

The survival of motoneurons (MNs) following severe nerve injuries at the Central/Peripheral Nervous Systems (CNS/PNS) interface is critical for achieving functional recovery. Among the experimental models used to investigate such lesions, the ventral root avulsion (VRA) stands out as one of the most robust and well‐characterized. This model leads to extensive spinal MN degeneration (Koliatsos et al. 1994; Oliveira and Langone 2000), accompanied by marked glial activation and synaptic stripping (Chai et al. 2000; Ribeiro et al. 2015), faithfully recapitulating the hallmarks of traumatic nerve damage.

Surgical reattachment of avulsed roots, particularly ventral root reimplantation (VRR) into the injured spinal cord, has demonstrated potential in promoting MN survival after VRA, supporting axonal regeneration, and improving functional recovery (Barbizan et al. 2014; Bigbee et al. 2008; Gu et al. 2004; Romeo‐Guitart et al. 2017). To further stabilize the reimplanted roots and optimize VRR outcomes, fibrin sealant (FS) can be used as a biocompatible tissue adhesive, providing stable root fixation while minimizing iatrogenic trauma associated with traditional suturing methods (Biscola et al. 2017). However, conventional FS products are typically derived from human blood components, raising concerns related to disease transmission, tissue necrosis, and seroma formation (Buchaim et al. 2019).

To overcome these limitations, researchers at the Center for the Study of Venoms and Venomous Animals (CEVAP, Brazil) developed an innovative fibrin sealant biopolymer (FSB) composed of a thrombin‐like enzyme from snake venom and bubaline‐derived fibrinogen (Ferreira et al. 2017). This innovative formulation offers superior adhesive capacity, reduced bleeding, shorter surgical times, and notable neuroprotective benefits—including enhanced synaptic preservation and improved motor and sensory functions (Barbizan et al. 2013).

Despite the clear benefits of surgical interventions for preserving MNs post‐injury, they are often insufficient for complete functional recovery. Thus, combining surgical repair with pharmacological agents has emerged as a compelling strategy to target multiple aspects of the injury response (Hu et al. 2023). Several compounds have demonstrated neuroprotective potential, including lithium (Fang et al. 2016; Fu et al. 2014), riluzole (Gloviczki et al. 2017), and dimethyl fumarate (Kempe et al. 2020). Additionally, various neurotrophic factors—such as brain‐derived neurotrophic factor (BDNF) (Xian et al. 2023), ciliary neurotrophic factor (CNTF) (Lang et al. 2005), glial cell‐derived neurotrophic factor (GDNF) (Eggers et al. 2020; Ruven et al. 2018), and fibroblast growth factor 2 (FGF‐2) (Araújo et al. 2017; Lima et al. 2024), have also been shown to counteract the deleterious effects of VRA.

Among emerging pharmacological candidates, the N, N dimethyltryptamine (DMT) has attracted increasing interest for its neuroprotective properties (Dean et al. 2019; Morales‐Garcia et al. 2020). DMT is an endogenous tryptamine alkaloid present in various plant species and synthesized in mammalian tissues, particularly in the brain and lungs (Barker 2022). DMT is also a major bioactive component of *Ayahuasca*, a traditional Amazonian preparation with a long history of spiritual and folk medicinal use (Frecska et al. 2016; James et al. 2022; McKenna and Riba 2018).

In this study, we investigated whether combining systemic DMT administration with VRR could synergistically enhance MN survival, attenuate glial reactivity, preserve synaptic integrity, and modulate the expression of genes involved in neurotrophic support and apoptotic regulation. By integrating a pharmacological neuroprotective agent with a targeted microsurgical repair strategy, our goal was to further elucidate DMT's therapeutic potential and contribute to the development of a multimodal strategy for treating traumatic nerve injuries and MN disorders.

## 2 Materials and Methods

### 2.1 Isolation and Chemical Characterization of N, N‐Dimethyltryptamine

DMT was isolated from *Mimosa tenuiflora* roots using a modified acid–base extraction protocol based on Gaujac et al. (2013). This method leverages the increased solubility of alkaloid salts in acidic aqueous media and the disruption of plant cell matrices under low pH, improving extraction efficiency.

**FIGURE 1:**
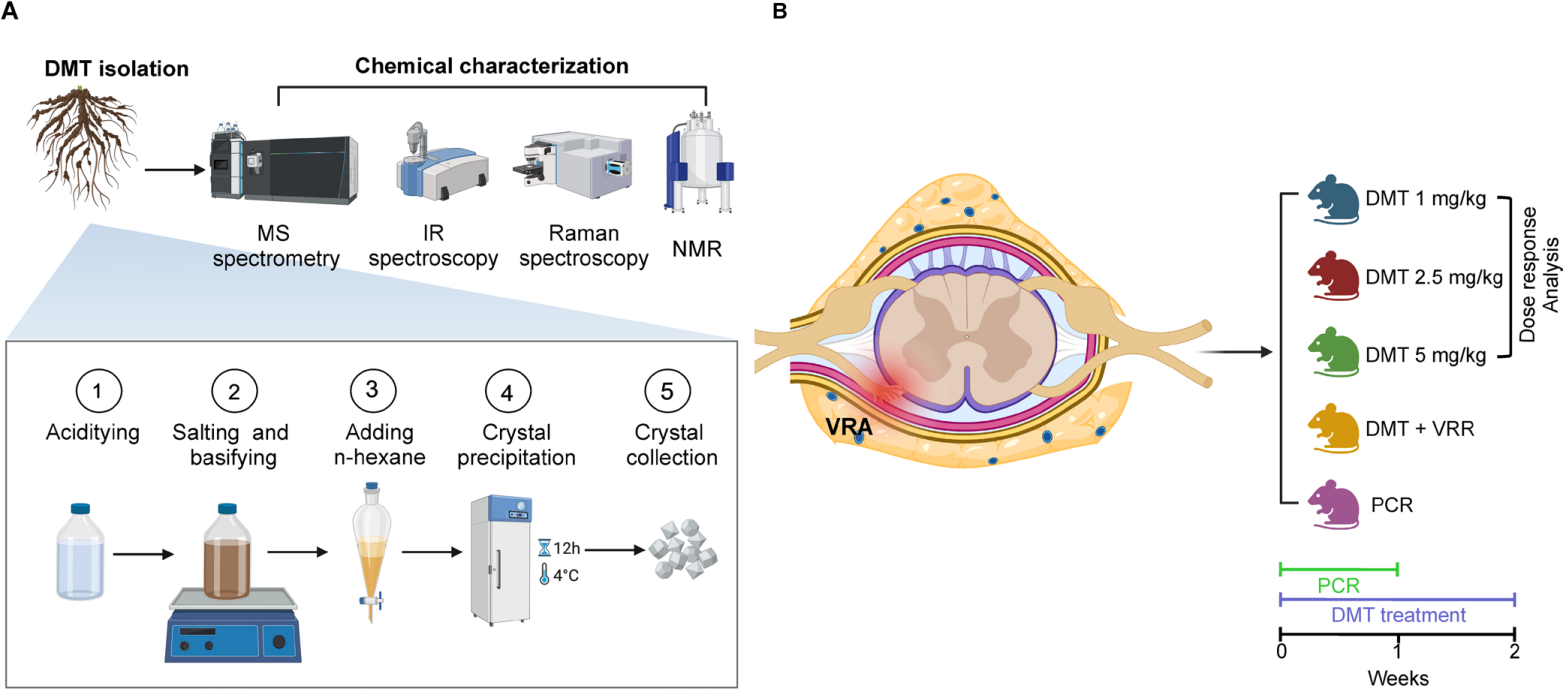
N, N dimethyltryptamine (DMT) isolation and experimental design. (A) DMT was extracted from *Mimosa tenuiflora* roots and structurally characterized using multiple analytical techniques. (B) Following extraction, a dose–response study was conducted in which animals underwent unilateral L4‐L6 ventral root avulsion (VRA) and received daily i.p DMT at 1, 2.5, or 5 mg/kg for 2 weeks (*n* = 5 rats per group). The optimal dose (1 mg/kg) was subsequently used in combination with ventral root reimplantation (VRR) for therapeutic assessment. For gene expression analysis (qPCR), animals were treated daily with DMT for 1 week prior to tissue collection. Created in https://BioRender.com.

The identity of the extracted DMT was confirmed using the following complementary analytical techniques (Figure 1):

#### 2.1.1 Liquid Chromatography Coupled to Mass Spectrometry (LC‐MS/MS)

LC–MS analyses were performed using a Thermo QExactive Orbitrap mass spectrometer coupled to a Waters BEH C18 column (2.1 × 50 mm × 1.7 μm). Data were acquired in Full Scan‐Data Dependent Acquisition (FS‐DDA) mode under positive ionization. The mobile phases consisted of water with 0.1% formic acid (phase A) and methanol with 0.1% formic acid (phase B), delivered at a flow rate of 200 μL/min. The column oven was maintained at 40°C throughout the run.

The instrument resolution was set to 70 000 (at *m/z* 200), with an acquisition range of *m/z* 50–750. A total of 5 μL of each sample was injected, and the overall analysis time was 25 min. Ionization parameters included a spray voltage of 3.5 kV and an Sl‐Lens RF level of 50 V.

For identity and purity confirmation, a certified DMT reference material was analyzed under the same LC–MS conditions. Specifically, DMT solution (catalog number: D102), 1.0 mg/mL in methanol (Cerilliant).

#### 2.1.2 Fourier‐Transform Infrared Spectroscopy (FTIR)

FTIR spectrum was recorded in the range of 400 to 4000 cm^−1^ using an Agilent Cary 660 FTIR spectrophotometer coupled with an attenuated total reflectance (ATR) sampling accessory.

#### 2.1.3 Raman Spectroscopy

Raman spectra were acquired using a HORIBA Scientific XploRA ONE Raman spectrophotometer coupled with a Jobin Yvon confocal microscope and a charge‐coupled device detector. Measurements were taken using a 785 nm laser at 37.6 mW power, with a 10× objective lens, 1‐s acquisition time, and 1 accumulation. The spectral range was 100–4000 cm^−1^.

#### 2.1.4 Proton Nuclear Magnetic Resonance ( ^1^H NMR)

^1^H NMR spectra were acquired using a Bruker Spectrospin Avance DPX‐200 spectrometer operating at 400 MHz. Measurements were conducted at 22°C using CDCl3 as solvent. All data were reported in terms of the chemical shift (δ, in ppm), multiplicity, and coupling constant (J, in Hz). The multiplicity of a particular signal was reported as s (singlet), d (doublet), t (triplet), or m (multiplet).

### 2.2 Animals and Experimental Design

A total of 50 female Lewis rats (7–10 weeks old, 180–200 g) were obtained from the Multidisciplinary Center for Biological Investigation (CEMIB/UNICAMP, Brazil). Animals were housed in pairs in polysulfone isolators (Alesco, Brazil) under controlled temperature, humidity, and light/dark cycle conditions, with food and water provided *ad libitum*. All experimental procedures were conducted in accordance with the guidelines of the Brazilian College for Animal Experimentation and were approved by the Institutional Committee for Ethics in Animal Use of the Institute of Biology, UNICAMP (CEUA/IB/UNICAMP; Protocol No. 5921‐1/2021). Special efforts were made to minimize animal numbers and reduce suffering during the experimental procedures.

Animals were arbitrarily assigned to experimental groups and subsequently labeled with unique numerical identifiers to ensure that data acquisition and analysis could be conducted in an unbiased fashion.

To estimate the number of animals per group, we used the sample size calculation for the comparison of two means, assuming equal variances. We adopted a two‐tailed test with power, an estimated standard deviation of *s* = 0.2, and a minimum difference of interest Δ. The formula applied was:

$$n=\left(\left(2^{*}s^{2*}{\left(z_{\left(1-\alpha /2\right)}+z_{\left(1-\beta \right)}\right)}^{2}\right)\right)/\Delta^{2}$$

With $z_{1-\alpha /2}=1.96$ and $z_{1-\beta }=1.2816$, we obtained *C* = 10.51 and therefore *n* = 3.36, rounded up to *n* = 4 animals per group. Considering possible losses during the experiment, we allocated 5 animals per group to ensure the planned statistical power.

The study was divided into three distinct experimental settings (*n* = 5/group/technique; see Figure 1):

- *DMT dose–response evaluation*: Animals underwent unilateral L4‐L6 VRA and received daily intraperitoneal (i.p) injections of DMT at doses of 1, 2.5, or 5 mg/kg for 2 weeks.
- *Combinatorial therapy assessment*: Following VRA, animals underwent VRR using the FSB and were treated with daily DMT (1 mg/kg, i.p) for 2 weeks.
- *Molecular analysis*: Animals were subjected to VRA followed by VRR (with FSB) and received daily DMT treatment (1 mg/kg, i.p) for 7 days. Spinal cord tissues were harvested from both the injured (ipsilateral) and uninjured (contralateral) sides. A group of intact, unoperated animals served as baseline controls.

DMT was stored under controlled conditions in sealed amber vials, and maintained at −20°C to prevent exposure to light, oxygen, and humidity. Fresh aliquots were prepared immediately before use by dissolving the compound in phosphate‐buffered saline (PBS) using an ultrasonic mixer (Fisher Scientific). To ensure compound stability throughout the study, quality control analyses using FTIR and Raman spectroscopy were performed 8 months after extraction, confirming that DMT retained its characteristic spectral profile with no detectable degradation products. DMT was administered 30 min post‐surgery, and PBS alone was used as the vehicle control.

### 2.3 Surgical Procedures

#### 2.3.1 VRA and Reimplantation With FSB

##### 2.3.1.1 Surgical Procedure

Animals were anesthetized with a combination of ketamine (Anasedan, 90 mg/Kg, i.p.) and xylazine (Dopalen, 10 mg/Kg, i.p). Anesthesia was maintained throughout the procedure with 2% isoflurane, and Bepanthen (Bayer, Germany) was applied to the eyes to prevent corneal desiccation. Animals were then subjected to unilateral laminectomy at T13‐L1 vertebrae followed by the avulsion of the lumbar ventral roots at L4, L5, and L6 spinal segments, according to the procedure already standardized by Oliveira and Langone (2000). Briefly, the spinal cord was exposed by laminectomy and a longitudinal incision was made to open the dural sac, the denticulate ligament was dissected, and ventral roots were carefully separated and avulsed with fine forceps (N° 4). Root stumps were rotated backwards to prevent spontaneous axon regeneration. The surgical site was then closed in anatomical layers (musculature, fascia, and skin).

##### 2.3.1.2 FSB Composition and Application

For root reimplantation procedures (VRR‐groups), avulsed roots were secured in their original anatomical position using a FSB developed and kindly donated by the CEVAP‐UNESP. The FSB comprised three sequentially applied components in a total volume of 6 μL: (i) 3 μL of fibrinogen‐rich cryoprecipitate obtained from *Bubalus bubalis* blood, (ii) 2 μL of calcium chloride diluent, and (iii) 1 μL of gyroxin, a thrombin‐like serine protease purified from *Crotalus durissus terrificus* venom (Barros et al. 2011).

The FSB components were isolated and characterized using standardized procedures at CEVAP. Further information on CEVAP is provided at https://youtu.be/CPcs4ity‐Uw. Buffalo‐derived fibrinogen was obtained from cryoprecipitate prepared from plasma of clinically healthy donor animals screened through vaccination, tuberculin testing, and serological analysis to ensure biosafety. Protein purity was confirmed by two‐dimensional electrophoresis as described by Abbade et al. (2021). Gyroxin was purified from lyophilized venom using low‐pressure liquid chromatography (Äkta Pilot—GE HealthCare Life Science, Sweden) with Unicorn 6.3 software for data acquisition. Gyroxin purity was confirmed by N‐terminal sequencing (EDMAN) and mass spectrometry (molecular mass ~30–34 kDa). The formulation details are protected under the patent BR1020140114327 (Ferreira et al. 2017). Upon component mixing, gyroxin addition triggered rapid polymerization, resulting in fibrin clot formation, stabilization, and visible coaptation of reimplanted roots.

#### 2.3.2 Postoperative Care and Verification

Animals subjected to surgical procedures were monitored in a temperature‐controlled recovery area until complete recovery from anesthesia, confirmed by the restoration of coordinated movements and normal responses to external stimuli. Throughout the experimental period, animals underwent comprehensive daily health monitoring to ensure postoperative welfare. These evaluations included systematic assessments of behavioral patterns, wound healing progression, body condition, posture, locomotor activity, and food and water intake. Particular attention was directed toward the detection of pain or distress‐related indicators, including piloerection, vocalization, abnormal gait or mobility, and changes in facial expressions assessed according to the Rat Grimace Scale described by Sotocinal et al. (2011).

In addition, gait function was specifically examined to detect motor impairments associated with the surgical intervention. Animals exhibiting normal hindlimb support or weight‐bearing on the affected limb after VRA were excluded from subsequent analyses, as these findings indicated incomplete avulsion. Only animals showing clear clinical signs consistent with complete L4–L6 root avulsion were included in the experimental groups. Approximately 4% of the animals were excluded and replaced based on these criteria. Furthermore, successful root avulsion and reimplantation were confirmed by direct visual inspection of the spinal cord during tissue collection.

Post‐operative analgesia was provided using tramadol hydrochloride (Germed Farmacêutica Ltda, Hortolândia/SP, Brazil) administered orally (5 mg/kg body weight) immediately after surgery and continued once daily for three additional days post‐operatively.

### 2.4 Specimen Preparation

For histological analysis, 2 weeks post‐VRA, animals were deeply anesthetized with ketamine (Anasedan, 90 mg/kg, i.p.) and xylazine (Dopalen, 10 mg/kg, i.p.), and euthanized via transcardial perfusion with PBS (pH 7.4), followed by 4% paraformaldehyde (PFA) in 0.1 M phosphate buffer (PB). The lumbar intumescence was carefully dissected, post‐fixed in 4% PFA overnight, and cryoprotected in a graded sucrose series (10%, 20% and 30% in 0.1 M PB, 24 h each at 4°C). Tissues were embedded in Tissue‐Tek O.C.T. (Sakura Finetec USA Inc., Torrance, USA) and frozen in n‐hexane at −32°C to −35°C, then stored at −20°C.

For quantitative RT‐PCR analysis, animals were anesthetized using the same anesthetic regimen, then transcardially perfused with PBS without fixative. Spinal cord segments encompassing the lesion site were carefully dissected, divided into ipsilateral and contralateral halves, frozen in liquid nitrogen, and stored at −80°C until further processing.

### 2.5 Histological and Immunofluorescence Analysis

#### 2.5.1 Motoneuron Survival

Serial transverse sections (12‐μm thick) of the L4‐L6 spinal cord segments were obtained using a cryostat (Leica CM1860). For MN quantification, fifteen equidistant slides per animal were processed for Nissl staining. Sections were immersed in 0.05% toluidine blue in distilled water for 30 s, rinsed in distilled water, dehydrated in graded alcohol series (70%, 80%, 90%, 100%), clearing in xylene, and coverslipped using Entellan (Merck, Darmstadt, Germany).

The lateral ventral horn was imaged under a light microscope at 20× and 40× magnification. Alpha MNs were identified based on their localization in lamina IX of Rexed of the spinal cord and quantified according to the following criteria: diameter greater than 30 μm, presence of a prominent nucleolus, and polygonal soma. To avoid double‐counting neurons in consecutive sections, counts were adjusted using the Abercrombie and Jhonson formula (Abercrombie and Johnson 1946). MN survival was expressed as the ipsilateral/contralateral ratio.

#### 2.5.2 Immunofluorescence

Transverse spinal cord sections were rinsed in 0.01 M PB (3× 5 min) and blocked for 1 h in 3% bovine serum albumin (BSA) at room temperature. Sections were incubated overnight at 4°C with the following primary antibodies: rabbit anti‐Iba1 (1:750, FUJIFILM Wako Pure Chemical Corporation Cat# 019‐19741, RRID:AB_839504); rabbit anti‐GFAP (1:750; Abcam Cat# ab7260, RRID:AB_305808); rabbit anti‐Synaptophysin (1:1000; Novus Cat# NBP2‐25170, RRID:AB_2814699). Following primary antibody incubation, sections were rinsed in 0.01 M PB (3× 5 min) and incubated with Alexa Fluor 488‐conjugated secondary antibody (1:250, Jackson ImmunoResearch Labs Cat# 111‐545‐008, RRID: AB_2338048) for 1 h at room temperature. All antibody dilutions were diluted in 0.1 M PB containing 1.5% BSA and 0.2% Triton X‐100. Slides were mounted in 0.1 M PB/glycerol.

#### 2.5.3 Imagen Acquisition and Quantification

Immunofluorescence images were acquired using a Leica DM5500B microscope equipped with a Leica DFC345FX camera. Images were acquired at 20× and 40× magnification and microscope settings were standardized across all experimental groups for each antibody.

Quantitative analysis was performed using ImageJ software (version 1.52; National Institute of Health, Bethesda, MD, USA) based on protocols described by Oliveira et al. (Kempe et al. 2020; Lima et al. 2024). Integrated pixel density was calculated as the ratio between ipsilateral and contralateral sites, using contrast enhancement and density slicing. Background correction was achieved using the maximum intensity projection function and a uniform threshold across all groups. Measurements were systematically performed across three alternated slides per animal.

For glial analysis, immunofluorescence images of Iba‐1 (microglia) and GFAP (astrocytes) were acquired at 20× magnification. To complement Iba‐1 immunolabeling and enable distinction between activated and surveying microglia, a comprehensive morphological assessment was performed. Microglial cells were classified into five distinct morphological phenotypes: types I and II were categorized as surveying or non‐activated, while types III, IV, and V were classified as activated. The morphological criteria were defined as follows: type I comprised cells exhibiting fewer than two processes; type II included cells displaying three to five short branches; type III characterized cells with more than five extended processes and compact soma; type IV consisted of cells with enlarged soma with attenuated and retracted processes; and type V designated cells with amoeboid soma accompanied by multiple short processes (Figure 6A).

Synaptic input onto MNs was assessed using 40× images of synaptophysin–immunolabeled sections. An elliptic template (∼80 μm^2^) was positioned over eight equidistant regions surrounding the soma of alpha MNs located in the dorsolateral nucleus, following a protocol already standardized (Araújo et al. 2017). The mean ipsilateral/contralateral ratio of pixel intensity was calculated for each animal.

### 2.6 qRT‐PCR Analysis

Seven days post‐VRA, animals were deeply anesthetized for spinal cord dissection. The L4‐L6 spinal segments were collected, and each segment was bisected into ipsilateral and contralateral halves, which were immediately frozen in liquid nitrogen. Total RNA was extracted using QIAzol Lysis Reagent (Qiagen—cat.no. 79306), and RNA concentration was determined using a nanophotometer (Implen, Germany).

Complementary DNA (cDNA) synthesis was performed using the High‐Capacity cDNA Reverse Transcription Kit (Applied Biosystems, catalog number: 4368814), using 2 μg of RNA according to the manufacturer's instructions.

Gene expression was assessed via real‐time quantitative PCR (qPCR) using TaqMan gene expression assays (Life Technologies). The analysis focused on transcripts related to neurotrophic factors (GDNF: Rn00569510_m1; FGF‐2: Rn00570809_m1; VGFA: Rn01511602_m1) and anti‐apoptotic genes (Bcl‐2: Rn99999125_m1; Bcl‐XL: Rn00437783_m1). Reactions were performed in triplicate using 1 μL of cDNA per sample, RNase‐free water, TaqMan Gene Expression Master Mix (2×; Life Technologies—PN 4 369 016), and TaqMan assays containing primers and hydrolysis probes. The reference genes GAPDH (Rn01755763_g1) and HPRT1 (Rn01527840_m1) were selected based on their expression stability and were detected using VIC‐labeled probes, while genes of interest were labeled with FAM fluorophore.

Amplification was performed on an Mx3005P real‐time PCR system (Agilent Technologies, Santa Clara, CA, USA) using the following thermocycling conditions: initial denaturation at 95°C for 10 min, followed by 45 cycles of 95°C for 15 s and 60°C for 1 min. Data were acquired using MxPro software (Agilent), and relative gene expression was calculated using the 2^−ΔΔ*Ct*^ method.

### 2.7 Statistical Analysis

Statistical analysis, including assessment for potential outliers, was performed using GraphPad Prism version 9.3.1 (GraphPad software LLC, USA). The software's built‐in outlier detection procedures did not identify any outliers in the datasets; therefore, all data points were retained and included in the analyses. Data distributions were first assessed for normality. Depending on the outcome, either parametric or non‐parametric tests were applied, with appropriate post hoc analysis. Statistical significance was accepted at *p* < 0.05. Results are presented as mean ± standard deviation (SD).

## 3 Results

### 3.1 DMT Chemical Characterization

DMT isolated from *Mimosa tenuiflora* roots was comprehensively characterized using a multi‐analytical approach, combining LC–MS/MS, FTIR, and Raman spectroscopies, and ^1^H NMR to ensure unambiguous identification and purity assessment.

### 3.2 LC–MS/MS Confirms DMT Identity Through Retention Time, Accurate Mass, and Characteristic Fragments

#### 3.2.1 Chromatographic Analysis

**FIGURE 2:**
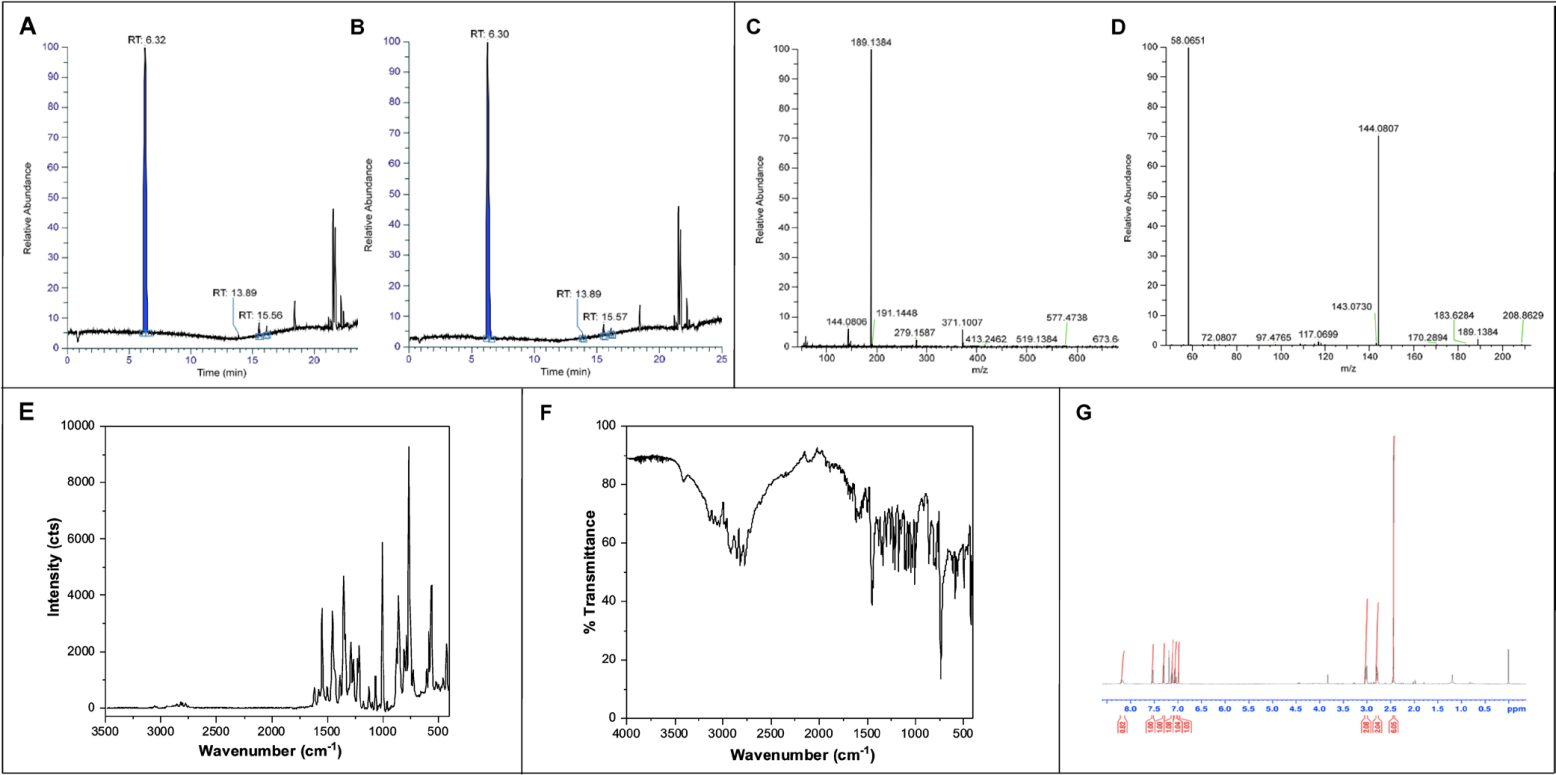
Chemical characterization of DMT. DMT was structurally characterized using complementary analytical techniques: (A, B) Liquid chromatography‐mass spectrometry (LC‐MS/MS) chromatograms showing comparable retention times (RT) for the reference standard (6.32 min) (A) and the isolated DMT sample (6.30 min) (B). (C, D) Mass spectrometric analysis revealed the molecular ion at *m/z* 189.1384 and a characteristic fragmentation pattern with prominent ions at *m/z* 58.06 and 144.08. (E) Raman spectrum highlighting vibrational modes associated with the indole nucleus and the dimethylamine moiety. (F) Fourier‐Transform Infrared spectroscopy (FTIR) spectrum displaying characteristic N—H stretching (~3400 cm^−1^), aromatic C—H stretching (~3050 cm^−1^), and indole ring vibrations. (G) Proton nuclear magnetic resonance (^1^H NMR) spectrum of DMT showing characteristic proton signals of the indole ring system (δ 7.0–7.6 ppm), methylene groups (δ 2.5–3.1 ppm), and N‐methyl groups (δ 2.3 ppm). Collectively, these analyses confirm the identity and purity of the isolated DMT.

To confirm the identity and purity of the analyte present in the laboratory‐derived extract, we analyzed a certified DMT reference standard under identical LC–MS conditions. Chromatographic analysis revealed that the reference standard displayed a single, well‐defined peak at retention time (RT) of 6.32 min, with a relative abundance of 97.89% (Figure 2A), corresponding to the dominant DMT signal. Under identical conditions, the extracted sample showed its main chromatographic peak at RT 6.30 min, with 98.1% relative abundance (Figure 2B). The minimal RT difference between the sample and standard (Δ*t* = 0.02 min) remains fully consistent with the acceptance limits defined by the SANTE/EC 2021/808 guidelines for LC–MS identity confirmation, which stipulate that RT deviations must not exceed ±0.1 min. The purity of the isolated DMT was determined to be ≥ 95%, calculated as the ratio of the peak area of the extracted DMT (1.030 × 10^10^) to the peak area of the standard (1.087 × 10^10^), multiplied by 100.

The close correspondence between both chromatographic profiles provides strong analytical evidence that the laboratory‐derived extract possesses a chemical composition consistent with that of the certified DMT reference standard.

#### 3.2.2 Mass Spectrometric Analysis and Fragmentation Patterns

Mass spectrometric evaluation of the extracted sample further corroborated its identity as DMT. Both the certified reference standard (Supporting Information, Figure S1) and the laboratory‐derived sample displayed the protonated molecular ion [M + H]^+^ at 189.1384, confirming the molecular formula C_12_H_16_N_2_ (MW: 188.27 g/mol) with sub‐ppm mass accuracy (Δm/z = 0.0001, 0.53 ppm) (Figure 2C).

The MS/MS fragmentation spectra (Figure 2D) exhibited key diagnostic ions characteristic of DMT. The base peak at m/z 58.0651 (100% relative abundance) corresponds to the dimethylammonium cation [C_3_H_8_N]^+^, formed by α‐cleavage of the ethylamine side chain, and is widely recognized as the most characteristic and informative fragment for DMT identification (Gaujac et al. 2013). A second major fragment was observed at m/z 144.0807 (≈70% relative abundance), corresponding to the indole methyleniminium cation [C_10_H_10_N]^+^, generated by neutral loss of dimethylamine (44 Da) from the protonated molecule. These ions, along with additional lower‐intensity fragments, collectively reflected the expected fragmentation pathways of DMT.

### 3.3 Vibrational Characterization of DMT by Raman and FTIR Spectroscopy

The Raman and FTIR analyses provided complementary vibrational fingerprints consistent with the expected molecular structure of DMT (Figure 2E,F). The Raman spectrum displayed several well‐defined vibrational features characteristic of the DMT molecular structure. Prominent Raman bands at 765, 858, 1003, 1433, and 1548 cm^−1^ correspond to indole ring breathing coupled with out‐of‐plane deformation, out‐of‐plane δCH bending of the pyrrole ring, symmetric ring breathing, in‐plane δNH₃^+^ deformation of the indole ring, and indole ring stretching vibrations, respectively. Additional bands at 1229, 1339, 1351, and 1385 cm^−1^ are associated with CH and CH₂ bending modes.

Consistently, FTIR analysis confirmed the presence of key functional groups through characteristic absorption bands. The FTIR spectrum exhibited diagnostic signals at 742, 810, 863, 1010, and 1050 cm^−1^, which are in strong agreement with previously reported vibrational assignments for DMT (Gaujac et al. 2013; Huertas‐Montoya et al. 2024; Vidak et al. 2015; Wu et al. 2022) and related tryptamines (Cañamares et al. 2019; Chuang and Chen 2009; daFonseca et al. 2017; Do et al. 2020; Gaigalas et al. 1998; Hussain and Pang 2015; Phung et al. 2019).

#### 3.3.1 NMR‐Based Structural Confirmation of DMT

^1^H NMR spectroscopy provided definitive structural confirmation of the extracted DMT (Figure 2G, Table S1). The ^1^H NMR spectrum (400 MHz, CDCl₃) displayed characteristic signals consistent with the DMT structure (Gaujac et al. 2013). Signal assignments were labeled from a to i, with their corresponding chemical shifts (δ, in ppm), multiplicities, and coupling constants (J, in Hz). Signal multiplicities were assigned as follows: s (singlet), d (doublet), t (triplet), and m (multiplet). The ^1^H NMR data δ in ppm were as follows: δ 2.46 (Ha, s, 6H); 3.0 (Hb, tdd, 2H, *J*_*bi*_ 0.67, *J*_*bc'*_ 7.69, *J*_*bc*_ 8.5 Hz), 2.78 (Hc, td, 2H, *J*_*bc'*_ 7.6, *J*_*bc*_ 8.5 Hz), 7.3 (Hd, ddd, 1H, *J*_*de*_ 8.07, *J*_*df*_ 1.17, *J*_*dg*_ 1.18 Hz), 7.05 (He, dd, 1H, *J*_*de*_ 8.07, *J*_*ef*_ 7.11 Hz), 7.12 (Hf, ddd, 1H, *J*_*dg*_ 1.17, *J*_*ef*_ 7.11, *J*_*fg*_ 7.8 Hz), 7.53 (Hg, ddd, 1H, *J*_*dg*_ 1.18, *J*_*fg*_ 7.8 Hz), 8.17 (Hh, s, 1H), 6.98 (Hi, d, 1H, *J* 0.67 Hz). Additional minor signals from residual solvent (CDCl_3_, 7.19 ppm) and water (3.82 ppm) were also detected. Importantly, the absence of additional significant signals or impurities in both the aromatic (6.5–8.5 ppm) and aliphatic (2–4 ppm) regions further corroborates the high purity of the isolated compound as determined by LC–MS/MS analysis.

Detailed atom‐to‐signal assignments and coupling analyses are provided in the Supporting Information.

Overall, the integrated analytical workflow provided a robust and unambiguous confirmation of DMT identity and quality. LC–MS/MS analysis established molecular identity through RT concordance with the certified reference standard, accurate mass determination of the protonated molecule, and the expected fragmentation pattern. Complementary vibrational spectroscopic data (Raman and FTIR) verified the presence of the indole core and the N, N‐dimethyl‐ethylamine substituent, while ^1^H NMR provided definitive structural confirmation, confirming the suitability of the laboratory‐driven DMT extract for subsequent neurobiological research applications.

### 3.4 Evaluation of Dose‐Dependent Effects of DMT on Neuroprotection

#### 3.4.1 DMT Enhances MN Survival Following VRA

**FIGURE 3:**
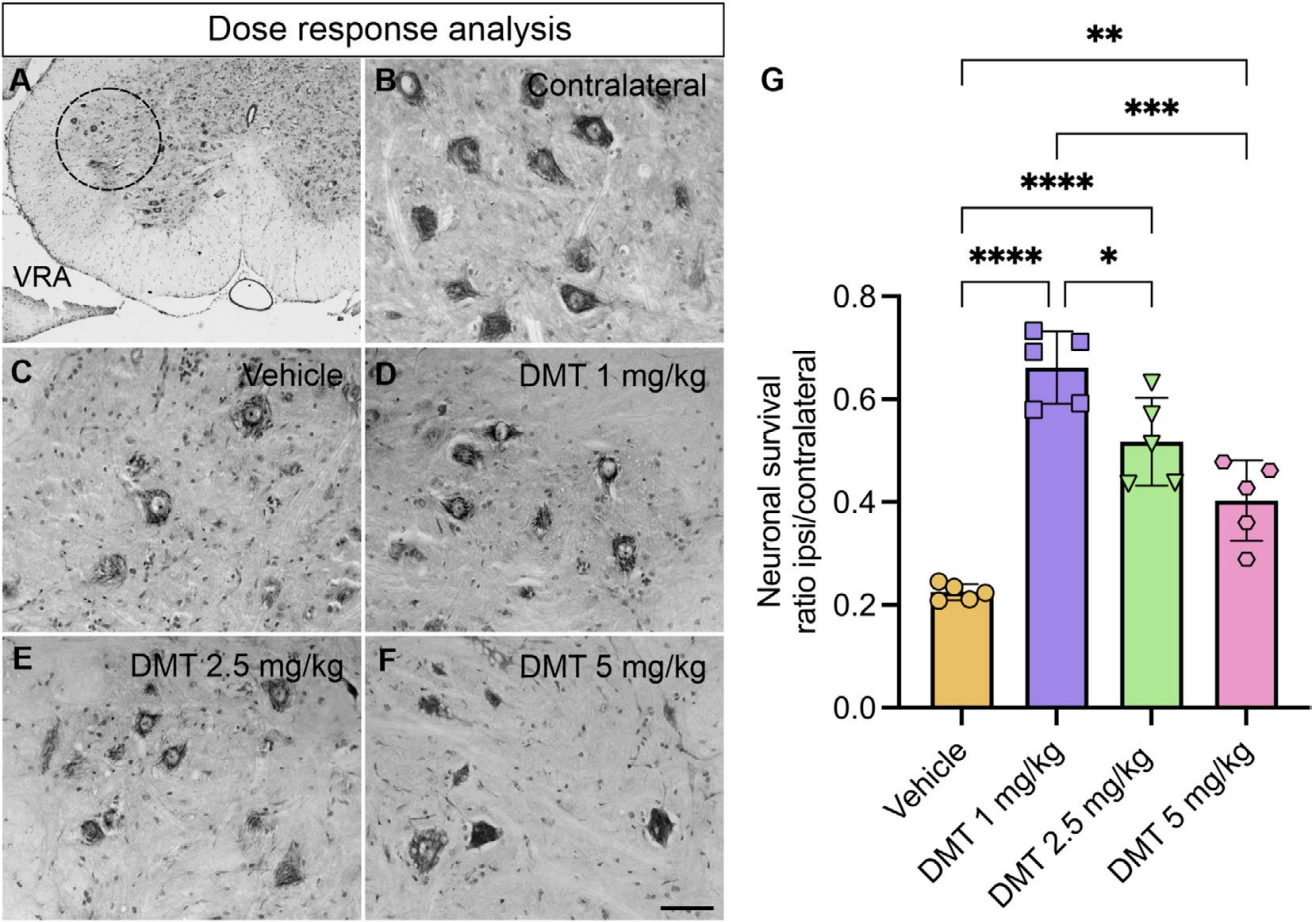
Motoneuron (MN) survival following unilateral VRA and DMT treatment at different doses. (A) Schematic representation of Rexed lamina IX (black circle) in the ventral horn of the spinal cord. (B–F) Representative Nissl‐stained sections from the contralateral (B) and ipsilateral (C–F) ventral horns across experimental groups. A marked reduction in MNs was observed ipsilateral to the lesion in the vehicle group, whereas all DMT treated groups showed a substantial preservation of MNs and improved cellular morphology. (G) Quantification of MN survival expressed as the ratio of MNs on the avulsed side relative to the contralateral ventral horn (mean ± SD; error bars represent SD). DMT treatment at 1 mg/kg and 2.5 mg/kg significantly enhanced MN survival compared with the 5 mg/kg dose (*n* = 5 rats per group). Scale bar = 100 μm. Statistical analysis was performed using one‐way ANOVA followed by Tukey's post hoc test (*p < 0.05; **p < 0.01; ***p < 0.001; ****p < 0.0001).

The neuroprotective efficacy of DMT was evaluated in a rat model of VRA. Histological evaluation demonstrated that proximal rhizotomy at the L4‐L6 spinal segments caused significant structural disruption and progressive degeneration of spinal MNs, evidenced by somatic atrophy, rounding of neuronal cell bodies, loss of polygonal morphology, and pronounced chromatolysis. These pathological changes were confined to the ipsilateral ventral horn, whereas the contralateral side maintained normal cytoarchitecture (Figure 3A–F).

Quantitative assessment at 2 weeks post‐injury revealed pronounced loss of MNs in the vehicle group, with only 22% of injured neurons surviving. In contrast, one‐way ANOVA confirmed that DMT treatment significantly improved MN survival in a dose‐dependent manner (*F*_(3,16)_ = 36.60, ****p < 0.0001). Tukey's post hoc analysis demonstrated that 1 mg/kg DMT provided the most robust neuroprotection, preserving 66% of MNs (****p < 0.0001), followed by 2.5 mg/kg (51%; ****p < 0.0001) and 5 mg/kg (40% **p = 0.0039). Furthermore, MN survival at 1 mg/kg was significantly higher than at both 2.5 mg/kg (*p = 0.0199) and 5 mg/kg (***p = 0.0001) (Figure 3G).

The ratios of surviving MNs in the ipsilateral side relative to the contralateral side were as follows:(mean ± SD): vehicle: 0.22 ± 0.01; DMT 1 mg/kg: 0.66 ± 0.07; DMT 2.5 mg/kg: 0.51 ± 0.03; DMT 5 mg/kg: 0.40 ± 0.07.

Importantly, surviving MNs in DMT‐treated groups displayed preserved morphological features, including large cell bodies, centrally located nuclei, and extensive dendritic arborization, closely resembling those observed on the uninjured contralateral side (Figure 3D–F).

#### 3.4.2 DMT Attenuates Injury‐Induced Glial Reactivity

**FIGURE 4:**
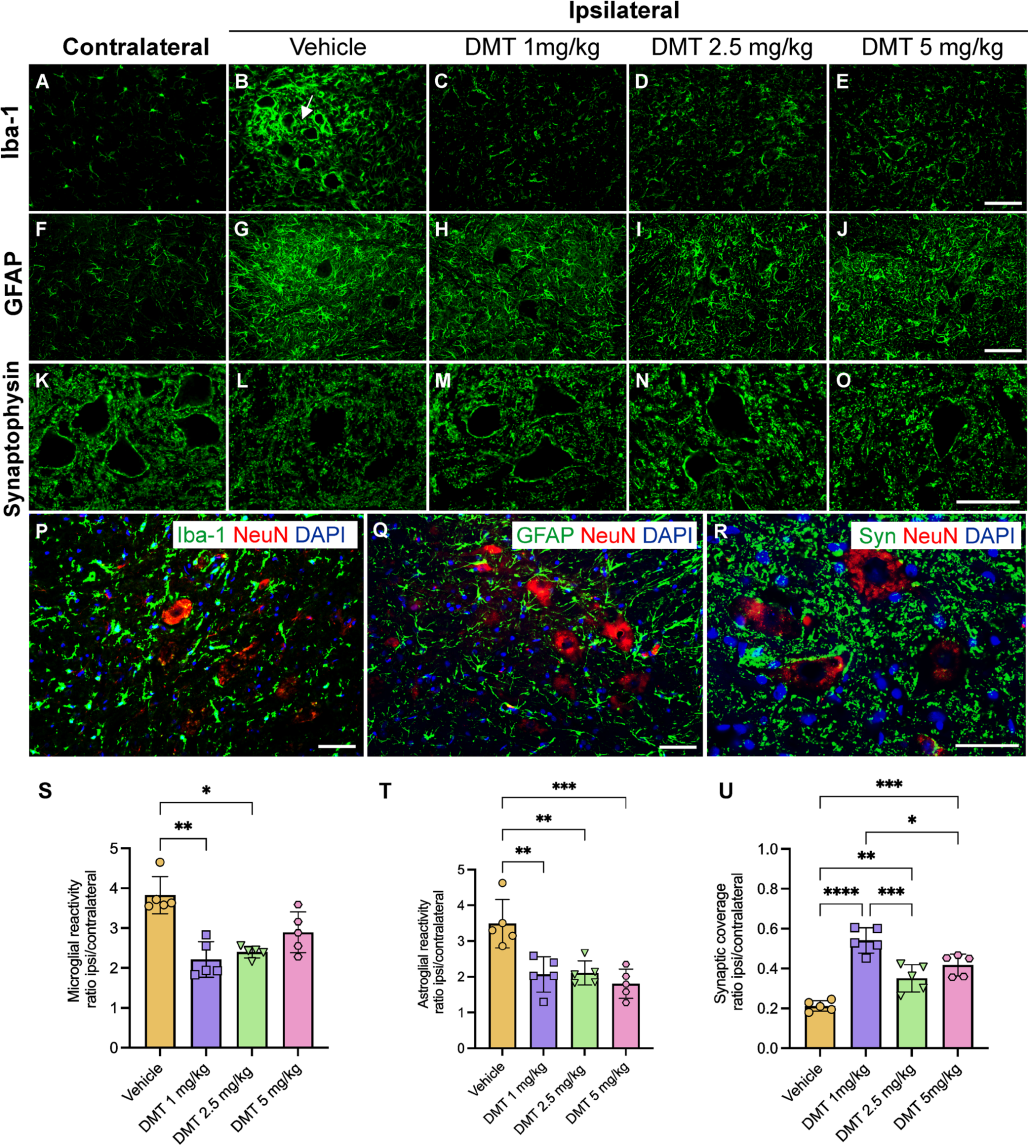
Glial responses and synaptic preservation following VRA and DMT treatment. (A–E, P) Representative immunofluorescence images showing microglial (Iba‐1), (F–J, Q) astrocytic (GFAP) and (K–O, R) synaptophysin labeling in Rexed lamina IX 2 weeks post‐VRA. Images correspond to both contralateral and ipsilateral ventral horns. Robust glial activation was evident in the vehicle group, whereas DMT‐treated groups displayed a marked attenuation of microglial and astrocytic reactivity, most notably at the 1 and 2.5 mg/kg doses. Activated microglia frequently surrounded avulsed MNs (white arrow in B). In parallel, DMT treatment significantly preserved synaptic coverage around MN somata compared to the vehicle group. Quantitative analysis of microglial (S) astrocytic (T) and synaptophysin (U) immunoreactivity are shown. Data are expressed as mean ± SD; error bars represent SD. Statistical analysis were performed using Kruskal‐Wallis test, followed by Dunn's post hoc test (S) and one‐way ANOVA followed by Tukey's post hoc test (T, U) (*p < 0.05; **p < 0.01; ***p < 0.001; ****p < 0.0001). (*n* = 5 rats per group). Scale bar = 100 μm.

To evaluate the effects of DMT on neuroinflammatory responses, immunofluorescence for Iba‐1 (microglia) and GFAP (astrocytes) was performed in Rexed's lamina IX. Analysis revealed that 2 weeks post‐VRA, the ipsilateral spinal cord exhibited pronounced glial activation, characterized by increased immunoreactivity and marked morphological alterations. Microglia displayed hypertrophic somata and perineuronal clustering, indicative of an activated phenotype, whereas astrocytes showed prominent hypertrophic processes (Figure 4B,G).

Immunofluorescence quantification demonstrated a significant overall effect of DMT treatment on microglial reactivity (Kruskal‐Wallis test, *H*_(3)_ = 12.54, *p* = 0.0057). Dunn's post hoc comparisons indicated that microglial immunoreactivity was significantly reduced in the 1 mg/kg (2.21 ± 0.44 vs. vehicle: 3.82 ± 0.46; **p = 0.0073), and 2.5 mg/kg groups (2.39 ± 0.14, *p = 0.0326), whereas the 5 mg/kg group did not differ significantly from the vehicle (2.89 ± 0.51, *p > 0.05) (Figure 4C–E,S).

Conversely, astrocytic reactivity exhibited greater sensitivity to higher DMT concentrations (Figure 4J). Statistical analysis by one‐way ANOVA (*F*_(3,16)_ = 11.67, *p* = 0.0003; Tukey's post hoc test) revealed that the highest dose of 5 mg/kg produced the greatest suppression of GFAP expression (1.80 ± 0.41 vs. vehicle: 3.48 ± 0.67; ***p = 0.0003) while both lower doses similarly attenuated astrocyte activation, showing comparable efficacy (1 mg/kg: 2.06 ± 0.49, **p = 0.0018; 2.5 mg/kg: 2.10 ± 0.34, **p = 0.0023) (Figure 4T).

#### 3.4.3 DMT Supports Synaptic Stability in Injured MNs

To assess synaptic preservation, synaptophysin immunolabeling was employed to assess synaptic density around MNs. Morphological analysis revealed well‐defined MN surfaces and distinct synaptic patterns among experimental groups. On the contralateral side, dense synaptic clustering with minimal spacing between inputs was consistently observed, reflecting normal synaptic organization (Figure 4K). In contrast, following VRA, the ipsilateral side exhibited marked synaptic terminal detachment, particularly in the vehicle group (Figure 4L). Remarkably, DMT treatment effectively preserved synaptic architecture, significantly mitigating synaptic loss (Figure 4M–O,R).

Synaptic density quantification further supported these findings. In the vehicle group, synaptic coverage over MN somata was drastically reduced, with synaptic inputs decreasing to 21%. In contrast, DMT treatment significantly preserved somatic synaptic inputs across all tested doses (one‐way ANOVA: *F*_(3,16)_ = 30.31, ****p < 0.0001; Tukey's post hoc test). The highest level of preservation was observed at 1 mg/kg, where 54% of synaptic inputs were maintained (****p < 0.0001), followed by 5 mg/kg (41%; ***p = 0.0001) and 2.5 mg/kg (35%; **p = 0.0055). Moreover, significant differences were detected between DMT 1 mg/kg and both DMT at 2.5 mg/kg (***p = 0.0003) and DMT 5 mg/kg (*p = 0.0138) (Figure 4U). Mean synaptic coverage values (mean ± SD) were as follows: vehicle: 0.21 ± 0.02; DMT 1 mg/kg: 0.54 ± 0.06; DMT 2.5 mg/kg: 0.35 ± 0.06; DMT 5 mg/kg: 0.41 ± 0.05.

Collectively, these findings underscore the multifaceted neuroprotective potential of DMT, including enhanced survival of avulsed MNs, attenuation of microglial and astroglial reactivity, and preservation of synaptic integrity. Among the doses tested, DMT at 1 mg/kg produced the most robust neuroprotective effects and was therefore selected as the optimal dose for subsequent experiments.

### 3.5 Combined Effects of Root Reimplantation and DMT on MN Survival, Glial Response and Synaptic Function

#### 3.5.1 Synergistic Neuroprotection by VRR and DMT Following Root Avulsion

Having established the neuroprotective efficacy of DMT and identified 1 mg/kg as the optimal dose, we next examined whether combining DMT with VRR using FSB could further potentiate neuroregenerative outcomes. Postoperative morphological inspection along with histological assessment of lumbar intumescence sections confirmed accurate reimplantation of the avulsed ventral roots into their original anatomical positions on the ipsilateral side.

**FIGURE 5:**
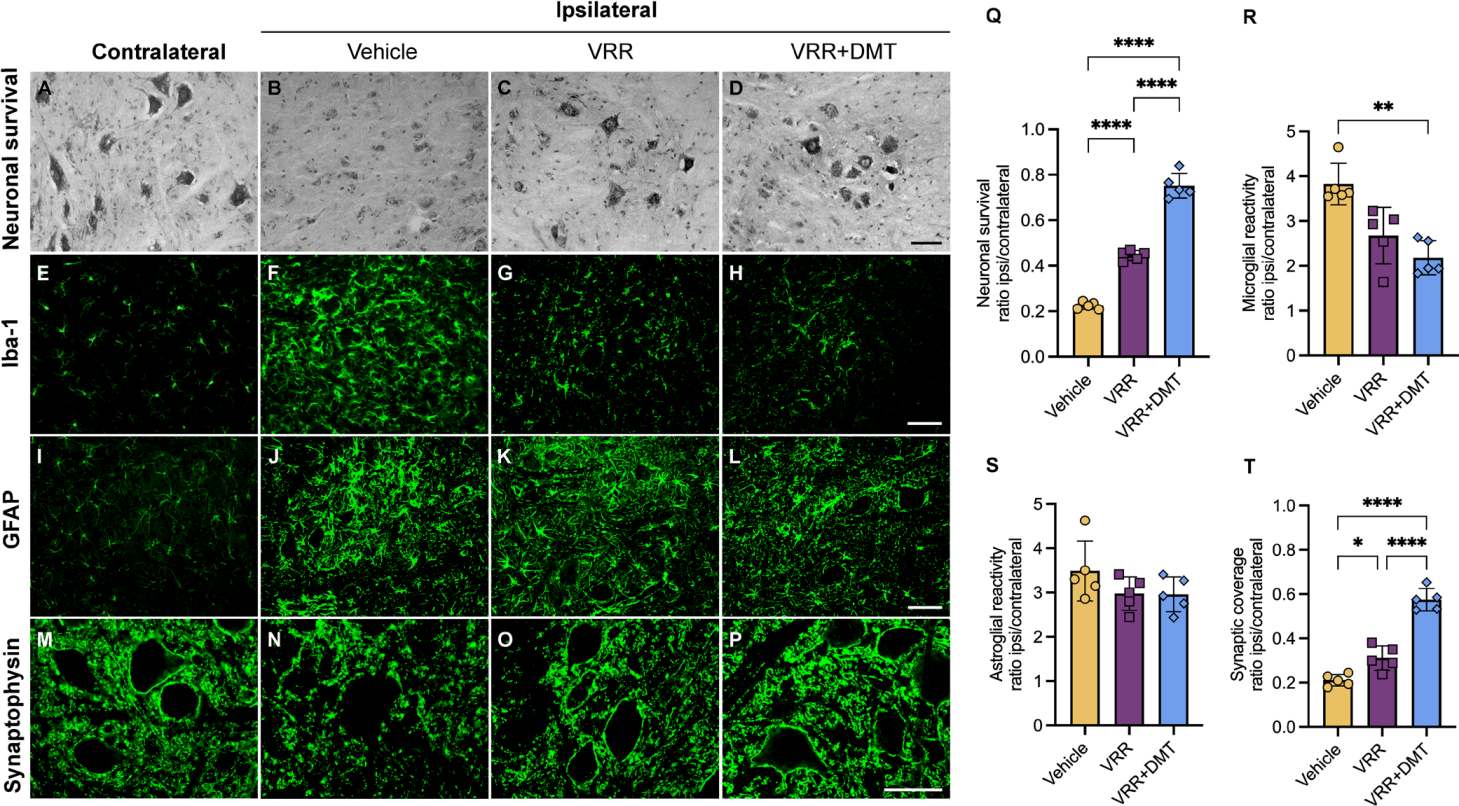
Effects of combined VRR and DMT treatment 2 weeks after VRA. Histological and immunofluorescence analysis demonstrated that the combinatorial approach significantly increased MN survival compared to both the vehicle and VRR‐alone groups (A–D). Microgliosis (E–H) was substantially reduced in the VRR and VRR + DMT groups relative to vehicle group, whereas astrogliosis (I–L) remained unaffected by treatment. Synaptic coverage (M–P) was increased following VRR‐alone and was further enhanced by the combined VRR + DMT treatment. Notably, the VRR + DMT group exhibited morphological and quantitative features closely resembling those observed on the contralateral (uninjured) side (M, O, P). (Q–T) Quantification of the ipsilateral/contralateral ratio (mean ± SD) for MN survival, microglial and astrocytic reactivity, and synaptic coverage across all experimental groups. Error bars represent SD. Statistical analysis was conducted using one‐way ANOVA followed by Tukey's post hoc test (*p < 0.05; **p < 0.01; ***p < 0.001; ****p < 0.0001) (*n* = 5 rats per group). Scale bar = 100 μm.

With the surgical accuracy verified, we proceeded to evaluate whether the combination of VRR and DMT translated into measurable improvements in MN preservation. Quantification of MN survival at 2 weeks post‐VRA demonstrated a significant treatment effect (one‐way ANOVA, *F*_(2,12)_ = 278.8, ****p < 0.0001). Tukey's post hoc test revealed that VRR alone significantly increased MN preservation to 44%, compared to 22% in the vehicle group (****p < 0.0001) (Figure 5C). Most notably, the combined VRR + DMT treatment produced a markedly greater neuroprotective effect, resulting in 75% MN preservation (Figure 5D). This improvement was statistically significant when compared to both the vehicle and VRR‐alone groups (****p < 0.0001 for both comparisons) (Figure 5Q). Corresponding mean survival ratios (mean ± SD) were: vehicle: 0.22 ± 0.01; VRR: 0.44 ± 0.02; VRR + DMT: 0.75 ± 0.05.

#### 3.5.2 Combined VRR and DMT Reduces Microgliosis but Not Astrogliosis

Microglial and astroglial responses, assessed by Iba‐1 and GFAP immunostaining respectively, exhibited differential patterns across treatment groups.

Assessment of microglial reactivity showed that only the VRR + DMT combination significantly attenuated microglial activation compared with the vehicle group (vehicle: 3.82 ± 0.46; VRR + DMT: 2.18 ± 0.38; **p = 0.0071; Kruskal‐Wallis test: *H*_(3)_ = 9.98; ***p = 0.0009; Dunns's post hoc test) (Figure 5H). In contrast, VRR alone produced a modest but non‐significant reduction in microglial reactivity (2.67 ± 0.63). No statistically significant difference was detected between the VRR‐alone and VRR + DMT groups (Figure 5R).

**FIGURE 6:**
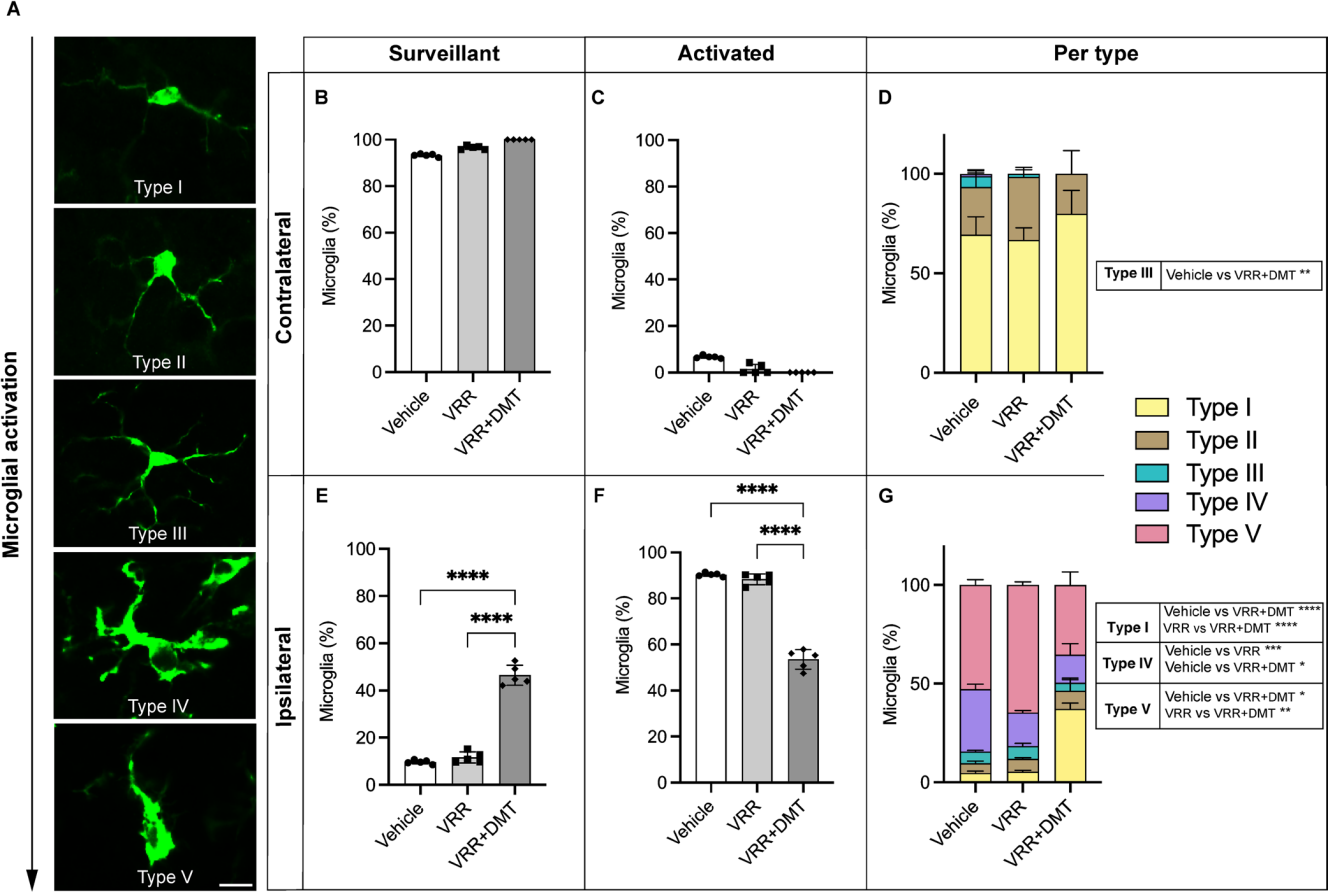
DMT treatment attenuates VRA‐induced microglial activation. (A) Representative photomicrographs of Iba‐1immunostained microglia classified according to their morphology into surveillant (types I and II) and activated (types III, IV, and V) phenotypes. Images were captured at 20× magnification; scale bar = 10 μm. (B, E) Distribution of surveillant microglial subtypes across experimental groups in the contralateral and ipsilateral spinal cord, respectively. (C, F) Distribution of activated microglia subtypes in the same regions. The VRR + DMT group exhibited significantly higher proportions of surveillant microglia compared to the vehicle and VRR‐alone groups. (D, G) Quantification of microglial subtypes in both contralateral and ipsilateral sides. Note the occurrence of type III microglia on the contralateral side in the vehicle and VRR groups. On the ipsilateral side, the VRR + DMT group demonstrated a marked reduction in activated microglia, predominantly type V cells, accompanied by a relative increase in type I microglia. Data are expressed as mean ± SD; error bars represent SD. Statistical analysis: (B, C, E, F) one‐way ANOVA with Tukey post hoc test. (D, G) Two‐way ANOVA with Tukey post hoc test (*p < 0.05; **p < 0.01; ***p < 0.001), (*n* = 5 rats per group).

These results are consistent with our detailed morphological classification of microglial phenotypes (Figure 6A). On the contralateral side, surveying microglia (types I and II) comprised approximately 97% of the microglial population across all experimental groups, with no significant intergroup differences (Figure 6B–D). In contrast, reactive microglia (type III) constituted a minor fraction (~3%) in the vehicle and VRR groups, but were entirely absent in the VRR + DMT group. This reduction was statistically significant compared to the vehicle group (type III) (**p = 0.0029**).

On the ipsilateral side, injury induced robust microglial activation, with approximately 75% of cells exhibiting reactive morphologies (types III, IV, and V; Figure 6E–G). Although reactive phenotypes predominated across all injured groups, the VRR + DMT combination significantly reduced the frequencies of both type IV and type V microglia compared to the vehicle (*p = 0.0149 and *p = 0.0122, respectively), and also significantly decreased type V microglia relative to VRR‐alone (**p = 0.0018). Furthermore, VRR‐alone also selectively reduced type IV microglia compared to the vehicle (***p = 0.0007). Importantly, the VRR + DMT group also exhibited a significant increase in the proportion of type I microglia compared to both vehicle and VRR‐alone groups (****p < 0.0001 for both), suggesting a phenotypic shift toward a more neuroprotective microglial profile in the combinatorial treatment group (Figure 6G).

Regarding astrocytic activation, GFAP immunoreactivity showed robust astrogliosis on the ipsilateral side in all groups (Figure 5J–L). Quantitative analysis did not detect statistically significant differences between DMT‐treated and vehicle groups (Figure 5S; ANOVA: *F*_(2,12)_ = 1.798, *p* = 0.2074; Tukey's post hoc test).

#### 3.5.3 Synaptic Protection Is Enhanced by Combined DMT + VRR Therapy

Building on the previously reported synaptoprotective effects of VRR (Barbizan et al. 2013) and the protective actions of DMT observed in our earlier analysis, we next evaluated their combined impact on synaptic integrity. Consistent with the preserved organization observed previously, synaptophysin immunostaining on the contralateral side exhibited a compact and continuous synaptic pattern surrounding MN somata (Figure 5M). Conversely, the ipsilateral side showed clear injury‐induced disruption, characterized by widened gaps between synaptic clusters. Notably, both VRR and VRR + DMT treatments attenuated this synaptic withdrawal, maintaining a denser and more cohesive synaptic arrangement than that observed in the vehicle group (Figure 5M–P).

Synaptic density quantification showed that VRR‐alone significantly enhanced synaptic coverage, increasing it from 21% in the vehicle group to 31% (*p = 0.0123) (Figure 5O). Remarkably, the VRR + DMT combination preserved 57% of synaptic inputs, representing a substantial improvement over both the vehicle and VRR‐alone groups (****p < 0.0001 for both comparisons) (Figure 5P). Synaptic coverage values (mean ± SD) were: vehicle: 0.21 ± 0.02; DMT 1 mg/kg: 0.31 ± 0.05; VRR + DMT: 0.57 ± 0.05. ANOVA: *F*_(2,12)_ = 83.62, ****p < 0.0001; Tukey's post hoc test.

Overall, the results presented here demonstrated that while DMT‐alone exhibited significant neuroprotective and immunomodulatory effects, markedly enhancing MN preservation, modulating glial reactivity, and improving synaptic integrity, these effects were further substantially amplified in the combinatorial approach with VRR. These findings support the premise that combinatorial therapeutic strategies can achieve superior outcomes following VRA injury.

#### 3.5.4 qRT‐PCR

To further elucidate the mechanisms underlying the observed neuroprotection, we conducted gene expression analysis of neurotrophic and anti‐apoptotic markers 7 days post‐injury.

**FIGURE 7:**
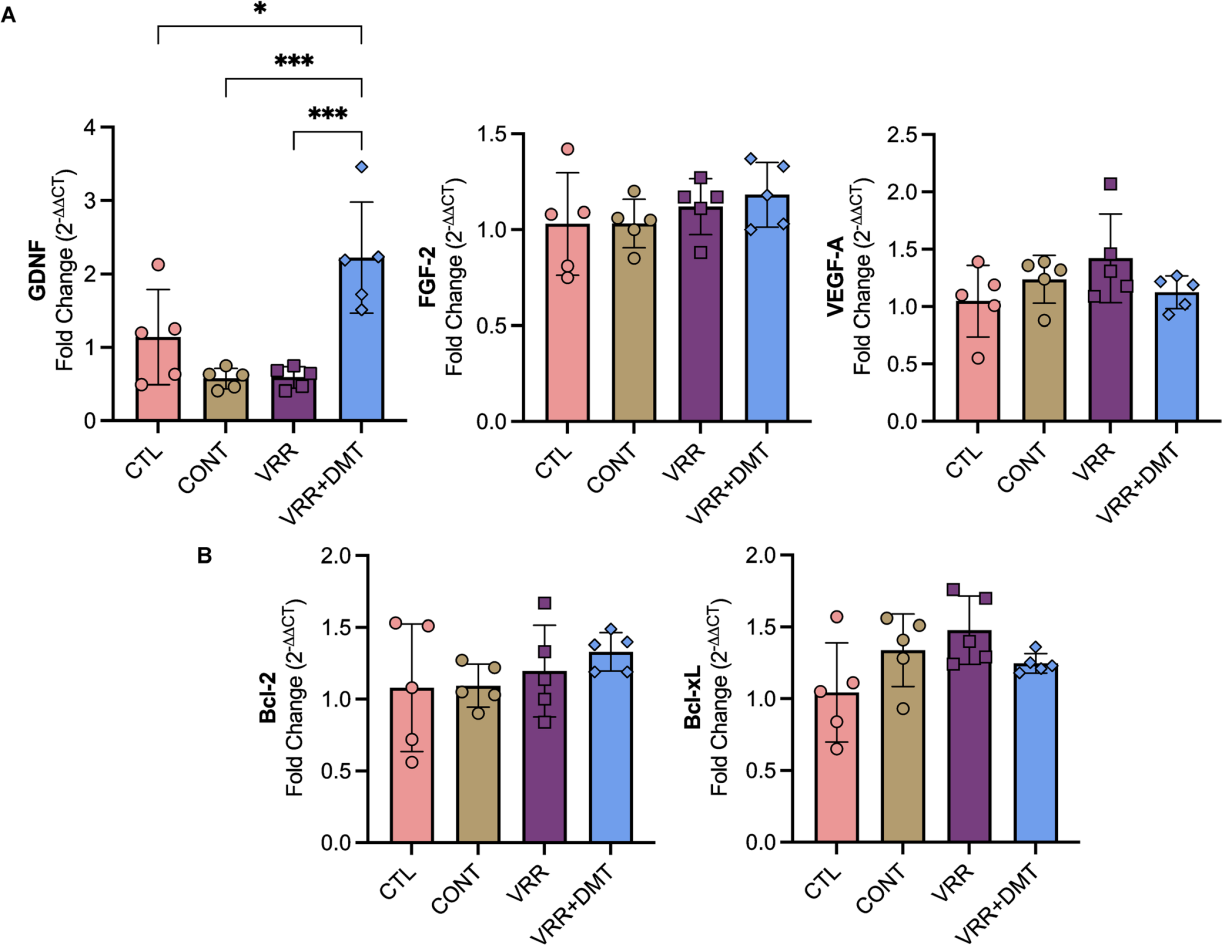
Gene expression levels of neurotrophic and anti‐apoptotic genes. (A) Expression analysis of neurotropic factors following VRR + DMT treatment revealed a significant upregulation of GDNF compared to the vehicle and VRR‐alone groups, while FGF‐2 and VEGF‐A expression levels remained unchanged. (B) Expression of anti‐apoptotic genes showed no significant differences among the experimental groups. Data are expressed as mean ± SD. Error bars represent SD. Statistical analysis was perfomed using one‐way ANOVA followed by Tukey's post hoc test (*p < 0.05; **p < 0.01***p < 0.001; ****p < 0.0001), (*n* = 5 rats per group). CONTRA, contralateral side; CTL, intact control; VRR + DMT, ventral root reimplantation + DMT treatment for 7 days; VRR, ventral root reimplantation.

Among neurotrophic factors analyzed, GDNF mRNA levels were significantly upregulated in the VRR + DMT group relative to both the contralateral control (CONT) and the VRR‐alone groups (***p = 0.0005 and ***p = 0.0006, respectively; one‐way ANOVA: *F*_(3,16)_ = 11.58, ***p = 0.0003; Tukey's post hoc test) (Figure 7). In contrast, GDNF transcript abundance was reduced in the intact control (CTL), CONT, and VRR groups, indicating that DMT potentiates a regenerative transcriptional program when combined with VRR. Although the VRR + DMT group also showed trends toward increased FGF‐2 and VEFG‐A mRNA expression, these differences did not reach statistical significance.

Analysis of the anti‐apoptotic genes Bcl‐xL and Bcl‐2 revealed no significant changes across experimental groups, suggesting that the observed neuroprotection may be mediated more by trophic and inflammatory modulation than by alterations in intrinsic apoptotic pathways.

## 4 Discussion

Treating injuries at the CNS/PNS interface remains a major clinical challenge (Kwon et al. 2004). Despite extensive research and numerous proposed therapeutic strategies, achieving functional regeneration has proven elusive (Carlstedt 2016; Mcdonald and Sadowsky 2002). This failure is primarily attributable to the complex and multifactorial pathophysiology of such injuries, which cannot be effectively addressed through single‐target interventions (Clifford et al. 2023). Consequently, combinatorial approaches capable of simultaneously modulating multiple pathological mechanisms may offer more promising outcomes (Sousa et al. 2020). Building on this premise, we investigated a novel combination therapy integrating surgical reimplantation with FSB and DMT, representing the first evaluation into the therapeutic potential of DMT in spinal cord injury.

In the initial phase of the study, DMT was isolated from *Mimosa tenuiflora* roots using an optimized acid–base extraction protocol. The identity of the compound was rigorously confirmed through comprehensive structural analyses, including mass spectrometry, FTIR, Raman spectroscopy, and NMR. These findings provided a solid foundation for subsequent experimental applications.

Subsequently, we assessed the potential neuroprotective properties of DMT using the VRA model, which induces profound MN degeneration by disconnecting MNs from their peripheral axons and associated glial cells (Koliatsos et al. 1994; Ruven et al. 2014). Consistent with previous reports (Barbizan et al. 2014; Cartarozzi et al. 2015, 2022; Kempe et al. 2023; Lima et al. 2024), we observed extensive neuronal loss following VRA, with approximately 22% MN survival 2 weeks post‐injury. Notably, DMT monotherapy significantly mitigated neuronal loss across all doses tested. However, the most neuroprotective dose was achieved at the lowest concentration (1 mg/kg), preserving up to 62% of injured MNs.

Even more compelling was the outcome observed with the combined therapy. When DMT was administered alongside VRR, MN survival increased to 75%, representing a substantial improvement over either intervention alone. This pronounced effect strongly suggests a synergistic interaction between these therapeutic approaches, likely attributable to complementary neuroprotective and regenerative mechanisms.

DMT's contribution to enhanced neuroprotection is supported by extensive in vitro and in vivo studies, demonstrating its ability to promote various aspects of neuronal development and repair, including neurogenesis, neuritogenesis, spinogenesis, and synaptogenesis (Morales‐Garcia et al. 2020; Szabó et al. 2021). These pro‐regenerative properties are complemented by DMT's demonstrated protective effects against multiple pathological processes implicated in neural injury, including hypoxia (Szabo et al. 2016), apoptosis (Nardai et al. 2020), and neuroinflammation (Borbély et al. 2022; Glynos et al. 2024).

In addition to the benefits conferred by DMT, the critical role of VRR cannot be overstated, especially considering that reimplantation of avulsed roots is essential for restoring anatomical continuity and facilitating sensorimotor recovery (Bigbee et al. 2008; Gu et al. 2004). VRR not only acts as a mechanical bridge promoting reconnection but also provides trophic support and enhances axonal regeneration (Barbizan et al. 2014; Chai et al. 2000; Gu et al. 2004). Moreover, previous studies have demonstrated that VRR promotes the expression of neurotrophic and anti‐inflammatory cytokines (Barbizan et al. 2013), further supporting its inclusion in multimodal therapeutic strategies.

While MN preservation provides compelling evidence of neuroprotection, modulation of glial responses offers additional insights into the mechanisms underlying therapeutic efficacy (Pottorf et al. 2022; Salvany et al. 2021). In this study, we assessed the activation profiles of both microglia and astrocytes, revealing treatment‐dependent modulations of glial reactivity. Our results showed that DMT administration, either alone or combined with VRR, effectively attenuated microglial activation, promoting a shift from reactive ameboid morphologies to surveillant, ramified phenotypes. Considering that sustained microglial reactivity contributes to synaptic stripping, neuronal damage, and impedes regeneration (Araújo et al. 2017; Barbizan and Oliveira 2010; Pottorf et al. 2022), the suppression of microglial activation by DMT likely represents a central mechanism underlying its neuroprotective effects.

These findings align with accumulating evidence showing that DMT exerts broad immunomodulatory actions through both receptor‐dependent and receptor‐independent mechanisms. In particular, DMT is an endogenous ligand of the sigma‐1 receptor (S1R), a pleiotropic chaperone that regulates cytokine release, inflammasome activity, and microglial phenotype (Szabo et al. 2014, 2016). S1R activation has been shown to suppress pro‐inflammatory M1‐like microglial states while promoting a shift toward M2‐like, pro‐regenerative phenotypes, characterized by increased production of anti‐inflammatory mediators such as IL‐10 and TGF‐β (Ooi et al. 2021; Szabó et al. 2021).

Beyond S1R signaling, DMT may also modulate neuroinflammatory responses through serotonergic pathways. As a potent agonist of the 5‐HT2A receptor, DMT can engage signaling pathways known to regulate innate immune responses, including suppression of TNF‐α and IL‐1β production in activated microglia and peripheral macrophages (Flanagan and Nichols 2018). Supporting this notion, recent studies demonstrate that psychedelic compounds structurally related to DMT, such as psilocybin and LSD, induce anti‐inflammatory shifts in microglial activation through 5‐HT2A‐dependent mechanisms (Kozlowska et al. 2021; Wiens et al. 2024; Inserra et al. 2021).

In parallel, with these receptor‐mediated effects, DMT attenuates NF‐kB‐driven pathways and reduces oxidative stress responses (Kelley et al. 2022), mechanisms closely linked to microglial‐driven secondary degeneration following CNS injury. NF‐kB is a master transcriptional regulator that controls the expression of pro‐inflammatory cytokines, chemokines, and enzymes involved in oxidative damage (Hayden and Ghosh 2012). Following CNS injury, persistent NF‐kB activation in reactive microglia perpetuates neuroinflammation and contributes to the expansion of tissue damage beyond the primary lesion site (Ghosh and Hayden 2008). Future studies in our laboratory will further evaluate these pathways to better understand the mechanisms influenced by DMT treatment.

Despite the robust microglial effects, DMT's influence on astrocytes followed a more nuanced and context‐dependent pattern. Under DMT monotherapy, astrogliosis was attenuated only at the highest dose tested (5 mg/kg), and this effect did not persist when DMT was combined with VRR. Although previous studies have shown that VRR independently reduces astrogliosis (Barbizan et al. 2013), one might expect additive or even synergistic interactions from the combined treatment. This discrepancy may reflect several possibilities: (i) the lower dose used in the combined protocol (1 mg/kg) may have been insufficient to modulate astrocytic responses; (ii) VRR may activate wound‐healing and tissue‐repair pathways that transiently amplify astrocytic reactivity as part of a physiological reparative program, potentially masking DMT's anti‐reactive effects; and (iii) the temporal dynamics of astrocyte activation may differ from those of microglia, such that peak astrocytic responses occur outside the time window examined in the present study.

Furthermore, in contrast to microglia, astrocytes are highly sensitive to subtle fluctuations in the extracellular environment, injury severity, and local signaling context (Yu et al. 2021). Their activation states are not only heterogeneous but also temporally and spatially regulated (Verkhratsky et al. 2023). Consequently, it is plausible that the timing, dosing, and molecular characteristics associated with DMT and VRR treatment influenced the net astrocytic outcome. To fully elucidate these dynamics, future studies should consider dose‐escalation paradigms, extended temporal analyses, and cell‐type specific molecular profiling of astrocyte subtypes (e.g., A1 neurotoxic vs. A2 neuroprotective phenotypes).

Nevertheless, the modest astrocytic modulation under VRR + DMT treatment should not be interpreted solely as a therapeutic limitation. Instead, it may indicate a beneficial preservation of essential astrocytic functions. Astrocytes are indispensable for CNS homeostasis, contributing to metabolic support, glutamate clearance, and blood–brain barrier integrity, processes essential for post‐injury recovery (Verkhratsky et al. 2023; Yu et al. 2021; Zhou et al. 2020). In this context, the ability of the combined therapy to selectively attenuate microglial activity while maintaining astrocytic functionality may represent a favorable therapeutic profile, offering anti‐inflammatory benefits without compromising vital neuroprotective roles.

This perspective becomes even more significant when considering the many roles of glial cells, which extend beyond neuroinflammation and glial scar formation. Accumulating evidence indicates that both astrocytes and microglia actively contribute to synaptic remodeling and stabilization following neuronal injury (Alvarez et al. 2020; Pottorf et al. 2022). These glial populations are actively involved in synaptic pruning (Kettenmann et al. 2013) and can dynamically interact with presynaptic terminals, either supporting their function or displacing them entirely, as originally described by Blinzinger and Kreutzberg (1968). While such glial‐mediated remodeling is essential for early regenerative processes, excessive or sustained glial activation may become maladaptive, leading to synaptic loss and impaired circuit reorganization (Salvany et al. 2021).

Within this framework, the ability of VRR + DMT to modulate glial reactivity likely constitutes one contributing factor to the enhanced synaptic preservation observed in our study. However, synaptic stability is governed by complex interconnected mechanisms, and glial regulation represents only one aspect of VRR + DMT's multifaceted effects. In line with findings of Ly et al. (2018), DMT may also stimulate synapse formation, increase dendritic arbor complexity, and promote dendritic spine growth, thereby contributing to structural plasticity and synaptic repair. VRR, in turn, may reinforce synaptic maintenance by restoring structural continuity and fostering neurotrophic support (Barbizan et al. 2013; Kempe et al. 2020; Lima et al. 2024), potentially explaining the superior synaptic preservation achieved with the combined therapy.

Although our results showed that synaptic coverage was not fully restored, the highest level of preservation (57% in the DMT + VRR group) was more than double that observed in the vehicle group (21%). This substantial improvement not only validates the efficacy of our multimodal therapeutic approach but also highlights the intricate interplay between inflammatory modulation, neuronal connectivity, and neuronal survival mechanisms. Therefore, these findings position synaptic preservation as a critical therapeutic target and potential biomarker for assessing treatment efficacy in CNS/PNS interface injuries.

To gain deeper insight into the mechanisms that may be driving this enhanced synaptic preservation and neuroprotection, we next examined the transcriptional landscape of axotomized spinal cord tissue. At the molecular level, axotomy triggers broad transcriptional reprogramming within the spinal cord, including both up‐ and down‐regulation of various genes (Liu and Wang 2020; Zhang et al. 2020). Among these, neurotrophic factors and apoptosis‐related genes have received particular attention due to their crucial roles in neuronal maintenance and survival following root and nerve injuries (Fogarty et al. 2019; Grumbles et al. 2009; Moujalled et al. 2021). Our analysis focused on the expression of key neurotrophic factors (GDNF, FGF‐2, VEGF‐A) and anti‐apoptotic markers (Bcl‐2, Bcl‐xL).

Strikingly, we observed a significant upregulation of GDNF exclusively in the VRR + DMT group. GDNF is among the most potent modulators of MN survival, synaptic maintenance, axonal regeneration, and mitochondrial function following injury (Cintron‐Colon et al. 2022; Eggers et al. 2020). Crucially, its therapeutic effectiveness depends not only on the presence of the ligand but also on the simultaneous upregulation of its receptors on injured MNs. After VRA, axotomized MNs increase transcription of the GDNF receptor complex—GFRα1 (GDNF family receptor alpha‐1) and the tyrosine kinase receptor RET, thereby enhancing their sensitivity to endogenous or treatment‐induced GDNF (Grumbles et al. 2009; Höke et al. 2000). This adaptive receptor upregulation effectively “primes” injured MNs, optimizing their trophic responsiveness and amplifying the neuroprotective impact of elevated GDNF levels. Importantly, this molecular modulation was strictly limited to GDNF. No significant changes were detected in FGF‐2, VEGF‐A, or in apoptosis‐related genes (Bcl‐2, Bcl‐xl), indicating that the combined therapy does not trigger a broad neurotrophic or pro‐survival transcriptional response. The absence of anti‐apoptotic gene upregulation, despite clear improvements in MN survival, further supports that the protective effects of VRR + DMT arise largely from enhanced GDNF signaling that may in turn influence the apoptotic machinery.

In summary, this study provides the first evidence of DMT's therapeutic potential, demonstrating its ability to promote neuroprotection, modulate glial responses, and preserve synaptic integrity—effects that appear to be mediated, at least in part, by enhanced GDNF signaling. Most importantly, our findings highlight the superior efficacy of combining DMT with VRR, supporting the premise that multimodal treatment strategies are better suited to address the complex pathophysiology of CNS/PNS interface injuries. These results open promising avenues for future therapeutic development targeting MN degeneration and regeneration.

## Author Contributions

**Paola Andrea Caro Aponte:** conceptualization, investigation, writing – original draft, methodology. **Edison Huertas Montoya:** methodology. **Italo O. Mazali:** methodology. **Alessandra Sussulini:** methodology. **Benedito Barraviera:** methodology. **Rui Seabra Ferreira Jr.:** methodology. **Luciana Politti Cartarozzi:** methodology, data curation. **Alexandre Leite Rodrigues de Oliveira:** conceptualization, funding acquisition, writing – review and editing, methodology, project administration.

## Funding

This work was supported by Fundação de Amparo à Pesquisa do Estado de São Paulo, 2018/05006‐0, 2021/02754‐9, 2023/02615‐4, 2023/16415‐7; Conselho Nacional de Desenvolvimento Científico e Tecnológico, 403159/2021‐0; Coordenação de Aperfeiçoamento de Pessoal de Nível Superior, Código de Financiamento 001.

## Conflicts of Interest

The authors declare no conflicts of interest.

## Supporting information


**Data S1:** Supporting Information.

## Data Availability

The data that support the findings of this study are available from the corresponding author upon reasonable request.
