# Supplementary material for: Combined Neuroprotective Effects of N,N‐Dimethyltryptamine and Ventral Root Reimplantation Following Spinal Root Avulsion in Rats
Source: J Neurochem. 2026 Jan 29;170(2):e70364. doi: 10.1111/jnc.70364 (PMC12856109; doi:10.1111/jnc.70364)
Supplement: Supplementary file 1 — Data S1: Supporting Information. [file JNC-170-0-s001.pdf]

COMBINED NEUROPROTECTIVE EFFECTS OF N, N DIMETHYLTRYPTAMINE AND VENTRAL ROOT REIMPLANTATION FOLLOWING SPINAL ROOT AVULSION IN RATS.

Paola Andrea Caro Aponte[1], Edison Huertas Montoya[2], Italo O. Mazali[2] Alessandra Sussulini[3], Benedito Barraviera[4], Rui Seabra Ferreira Jr.[4], Luciana Politti Cartarozzi[1], Alexandre Leite Rodrigues de Oliveira[1].

[1] Laboratory of Nerve Regeneration, University of Campinas (IB/UNICAMP), Brazil.

[2] Functional Materials Laboratory, Institute of Chemistry, University of Campinas (UNICAMP), Brazil.

[3] Laboratory of Bioanalytics and Integrated Omics (LaBIOmics), Institute of Chemistry, University of Campinas (UNICAMP), Brazil.

[4] Center for the Study of Venoms and Venomous Animals (CEVAP), São Paulo State University (UNESP), Botucatu, SP, Brazil.

*** Correspondence:**
alroliv@unicamp.br

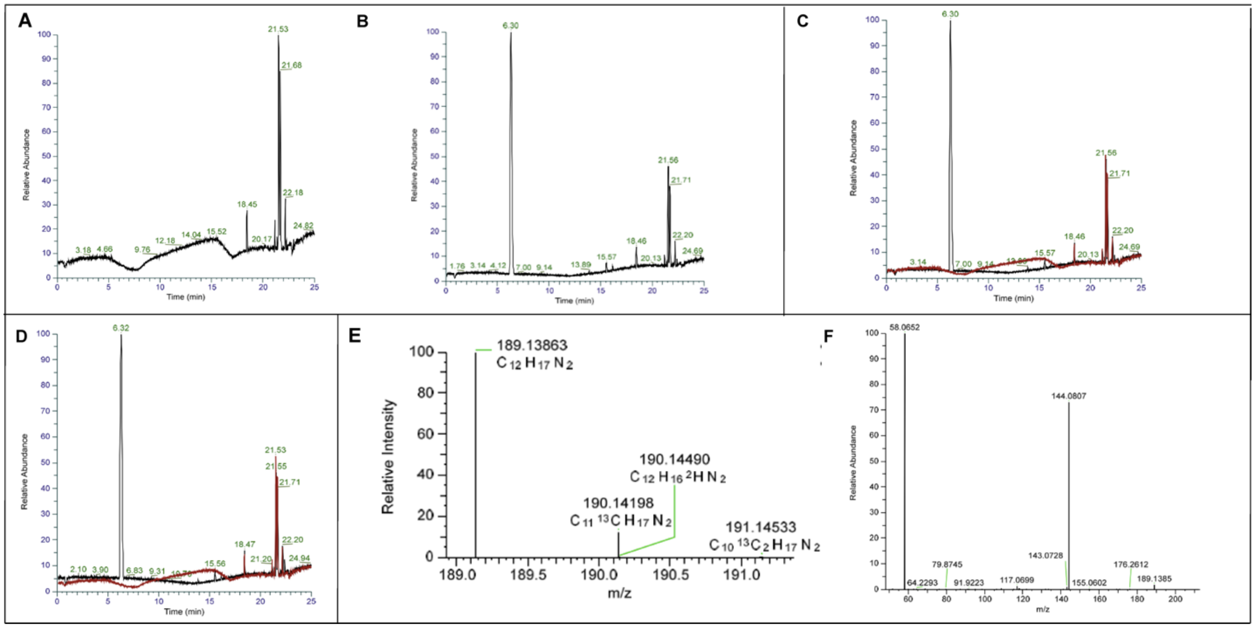

Figure S1. (A) Total ion chromatogram (TIC) of the blank sample. (B) TIC of the laboratory-extracted DMT showing the major DMT peak at 6.30 minutes. (C) Overlay of the extracted DMT (black) with the blank (in red) for comparison. (D–F) Analytical characterization of the certified DMT reference standard. (D) TIC of the reference standard, showing the main peak at 6.32 minutes, overlaid with the blank (in red). (E) High-resolution mass spectrum, showing the simulated protonated molecular ion at m/z 189.138. (F) MS/MS fragmentation spectrum, highlighting the principal product ions at m/z 58.0652 and 144.0807.

<table>
<tr><th>Graphic assignment</th><th>Position</th><th>δ (ppm) findings</th><th>δ (ppm) reported</th><th>Multiplicity</th></tr>
<tr><td rowspan="11">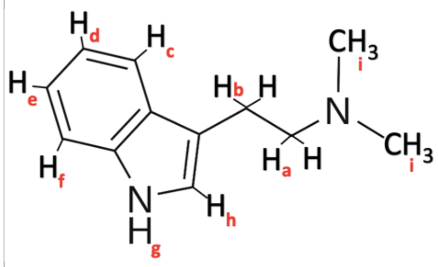
</td><td>Ha</td><td>3.0 2H</td><td>3.0 2H</td><td>Triplet-roof effect</td></tr>
<tr><td>Hb</td><td>2.78 2H</td><td>2.69 2H</td><td>Triplet-roof effect</td></tr>
<tr><td>Hc</td><td>7.3 1H</td><td>7.14 1H</td><td>Doublet</td></tr>
<tr><td>Hd</td><td>7.05 1H</td><td>7.63 1H</td><td>Triplet</td></tr>
<tr><td>He</td><td>7.12 1H</td><td>7.18 1H</td><td>Triplet</td></tr>
<tr><td>Hf</td><td>7.53 1H</td><td>7.14 1H</td><td>Doublet</td></tr>
<tr><td>Hh</td><td>6.98 1H</td><td>6.98 1H</td><td>Singlet</td></tr>
<tr><td>Hg</td><td>8.17 1H</td><td>8.49 1H</td><td>Singlet</td></tr>
<tr><td>Hi</td><td>2.46 6H</td><td>2.39 6H</td><td>Singlet</td></tr>
<tr><td>$CDCl_3$</td><td>7.19</td><td></td><td>singlet</td></tr>
<tr><td>H_2O</td><td>3.82</td><td></td><td>singlet</td></tr>
</table>

Table S1. ^{1}H NMR spectroscopic characterization and structural assignment of extracted DMT. The molecular structure (left) shows the graphic assignment of protons (Ha-Hh) corresponding to their respective positions in the DMT molecule. Chemical shifts (δ, in ppm) are reported for both the experimental findings and literature values (Gaujac et al., 2013), along with multiplicity patterns observed in $CDCl_3$. The close correspondence between experimental and reported values ($\Delta\delta < 0.1$ ppm for all signals) confirms the structural identity of the extracted compound.
